# Prognostic impact of peritoneal washing cytology in patients with biliary tract cancer

**DOI:** 10.1007/s00423-024-03233-y

**Published:** 2024-01-22

**Authors:** Tatsuaki Sumiyoshi, Kenichiro Uemura, Ryuta Shintakuya, Kenjiro Okada, Kenta Baba, Takumi Harada, Masahiro Serikawa, Yasutaka Ishii, Shinya Nakamura, Koji Arihiro, Yoshiaki Murakami, Shinya Takahashi

**Affiliations:** 1 Department of Surgery, Graduate School of Biomedical and Health Science, Hiroshima University, 1‐2‐3 Kasumi, Minami‐ku, Hiroshima, 734‐8551 Japan; 2 Department of Gastroenterology and Metabolism, Graduate School of Biomedical and Health Science, Hiroshima University, Hiroshima, Japan; 3 Department of Anatomical Pathology, Hiroshima University, Hiroshima, Japan; 4 Digestive Disease Center, Hiroshima Memorial Hospital, Hiroshima, Japan

**Keywords:** Biliary tract cancer, Peritoneal washing cytology, Carbohydrate antigen 19–9

## Abstract

**Purpose:**

To elucidate the clinical significance of peritoneal washing cytology (PWC) in patients with resectable biliary tract cancer (BTC).

**Methods:**

Clinical data of patients with BTC, who received PWC at curative intent surgery from March 2009 to December 2021, were retrospectively analyzed. Eligible patients were stratified into two groups according to positive or negative PWC. Recurrence-free survival and overall survival were compared between the two groups. Independent factors associated with positive PWC were investigated using multivariate analysis.

**Results:**

Among the 284 patients analyzed, all 53 patients with ampullary carcinoma showed negative PWC and these patients were excluded. Among the remaining eligible 231 patients, 41 patients had intrahepatic cholangiocarcinoma, 55 had gall bladder carcinoma, 72 had hilar cholangiocarcinoma, and 63 had distal cholangiocarcinoma. Eleven (4.8%) patients had positive PWC, and 220 (95.2%) had negative PWC. The median recurrence-free survival in the positive and negative PWC groups were 12.0 *vs.* 60.7 months (*p* = 0.005); the median overall survival times were 17.0 *vs.* 60.6 months (*p* = 0.008), respectively. Multivariate analysis revealed that serum carbohydrate antigen 19–9 level over 80 U/mL and multiple lymph node metastasis were independently associated with positive PWC (odds ratio [OR]: 5.84, *p* = 0.031; OR: 5.28, *p* = 0.021, respectively).

**Conclusion:**

Patients with positive PWC exhibited earlier recurrence and shorter survival times compared with those with negative PWC.

**Supplementary Information:**

The online version contains supplementary material available at 10.1007/s00423-024-03233-y.

## Introduction

Biliary tract cancer (BTC) is an aggressive tumor, and most patients exhibit advanced disease at presentation [1]. The incidence of cholangiocarcinoma has increased progressively worldwide over the past few decades [2–5], and surgical resection remains the only curative treatment option for this tumor [6–11]. As an important tool for determining the surgical indication, peritoneal washing cytology (PWC) has been extensively used in other malignancies. Positive PWC is regarded as a poor prognostic factor in the guidelines for gastric and ovarian cancer [12–15]. For pancreatic cancer, Ferrone et al. reported that patients with positive PWC had a similar survival rate as that of patients with stage IV disease [16], and this result is mentioned in the National Comprehensive Cancer Network guideline. However, the clinical significance of PWC in BTC remains unclear, because, to date, only a few studies have investigated this problem [17–19]. PWC has been routinely performed during curative-intent surgery at author’s institution, and this study aimed to elucidate the clinical significance of positive PWC status in patients with resectable BTCs.

## Methods

### Study design

In the author’s institution, PWC has been routinely performed at laparotomy in patients with BTC. The clinical data of eligible patients were collected through a retrospective review of medical records. The study was approved by the Ethics Review Board of Hiroshima University Hospital. Informed consent was waived due to the retrospective nature of the study. All procedures performed were in accordance with the ethical standards of the 1964 Helsinki Declaration and its later amendments or comparable ethical standards.

### Patient selection

**Fig. 1:**
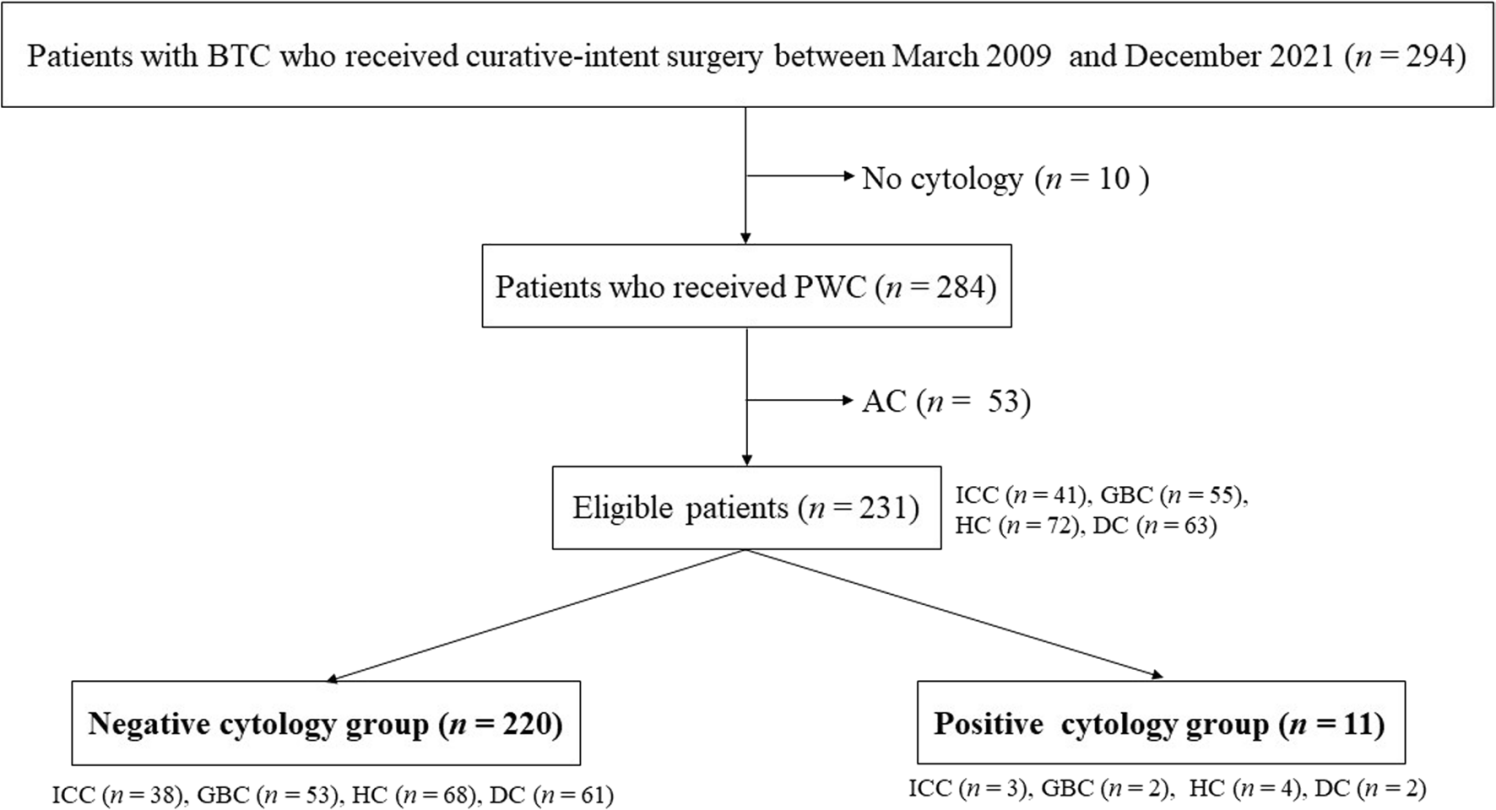
Patient flow chart. BTC, biliary tract cancer; PWC, peritoneal washing cytology; ICC, intrahepatic cholangiocarcinoma; GBC, gall bladder carcinoma; HC, hilar cholangiocarcinoma, DC, distal cholangiocarcinoma; AC, ampullary carcinoma

Eligible patients were defined as those with BTC who received collection of PWC at curative-intent surgery between March 2009 and December 2021 (Fig. 1). Four BTCs of intrahepatic cholangiocarcinoma (ICC), gall bladder carcinoma (GBC), hilar cholangiocarcinoma (HC), and distal cholangiocarcinoma (DC) were included [19–21]. Ampullary carcinoma (AC) was excluded according to the previous multi-institutional retrospective study [19].

### Cytology samples

PWC samples were collected during surgery according to the Japanese General Rules for the Study of Pancreatic Cancer [22]. Patients who had peritoneal dissemination at laparotomy were excluded from this analysis. Immediately after laparotomy, 100 mL of saline was injected into the pelvis, and as much fluid as possible was collected using a syringe. Subsequently, curative-intent surgery was performed, irrespective of the result of PWC. Two experienced pathologists who specialized in biliopancreatic malignancy confirmed the diagnosis. Papanicolaou and Gimza stains were used in cytology. Class 4 or 5 in cytology were diagnosed as positive PWC.

### Surgery

The standard surgery for HC and ICC with hilar invasion is major hepatectomy (hemihepatectomy and caudate lobectomy or trisegmentectomy and caudate lobectomy) concomitant with extrahepatic bile duct resection (BDR) and bilioenteric anastomosis. The standard surgery for DC is pylorus-preserving pancreaticoduodenectomy (PPPD). Hepatectomy is usually performed on peripheral type ICC. Hepatopancreatoduodenectomy (HPD) is performed in patients with extensive tumor invasion along the biliary tract. Regional lymphadenectomy and para-aortic lymph node sampling were performed in all cases.

### Outcome measures

**Fig. 2:**
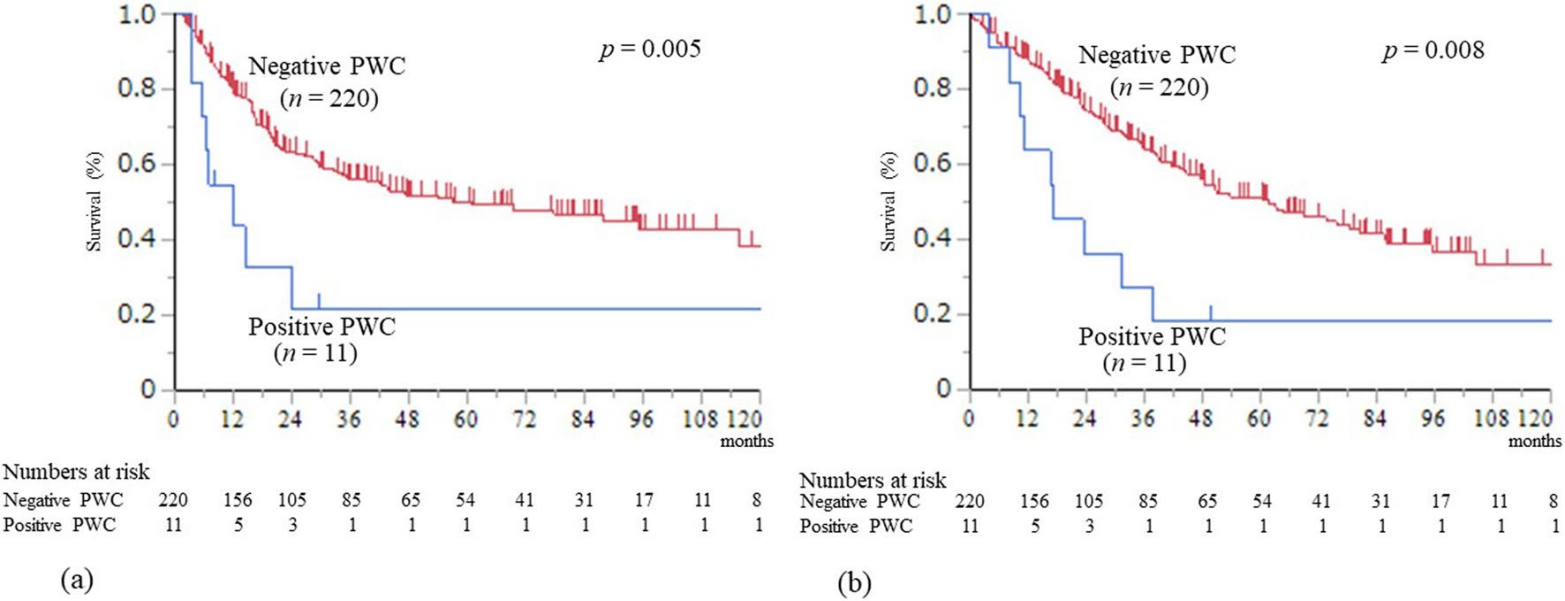
Survival curves for the positive and negative PWC groups. **a** Recurrence-free survival curves after surgery. **b** Overall survival curves after surgery. PWC, peritoneal washing cytology

**Fig. 3:**
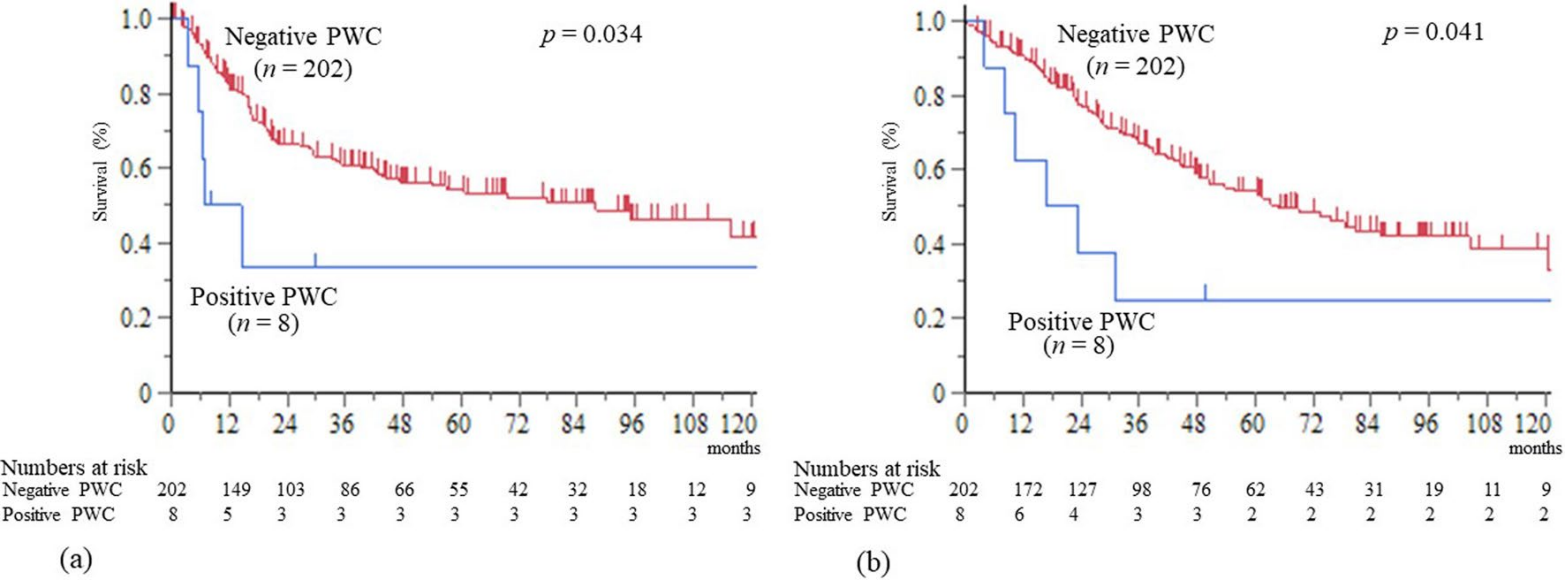
Survival curves in M0 cases. **a** Recurrence-free survival curves after surgery. **b** Overall survival curves after surgery. PWC, peritoneal washing cytology

Eligible patients were stratified into two groups according to positive or negative PWC. A comparison of the clinicopathological features (Table 1) and postoperative courses (Table 2) between positive and negative PWC groups was conducted. Union for International Cancer Control 8th edition was used for TNM classification. Carbohydrate antigen 19–9 (CA19-9) value was measured when serum total bilirubin level became under 3.0 mg/dL. The clinical courses of patients in the positive PWC group were investigated (Table 3). Recurrence-free survival (RFS) and overall survival (OS) were compared between the two groups (Fig. 2). RFS and OS were also compared in M0 cases to evaluate the prognostic impact of positive PWC alone (Fig. 3). Further, RFS and OS were compared between the four BTCs to evaluate the influence of type of carcinoma (Supplemental Fig. 1). The preoperative factors independently associated with positive PWC were investigated using multivariate analysis (Table 4).

**Table 1:** Clinicopathological features of the positive and negative peritoneal washing cytology groups

	Positive PWC (*n* = 11)	Negative PWC (*n* = 220)	*P* value
Preoperative factors			
Age (median, range)	71(46–85)	72 (18–92)	0.899
Gender (male / female), *n*	4/7	138/82	0.080
BMI (median, range)	23.3 (17.4–27.4)	22.9 (15.5–30.1)	0.837
Type of carcinoma, *n* (%)			0.755
ICC GBC HC DC	3 (27.3%) 2 (18.2%) 4 (36.4%) 2 (18.2%)	38 (17.3%) 53 (24.1%) 68 (30.9%) 61 (27.7%)	
Preoperative biliary drainage			0.099
EBD /PTBD / No drainage, *n*	9/1/1	147/4/69	
Laboratory data (median, range)			
Hb (g/dL)	11.6 (8.6–13.4)	11.9 (7.0–17.5)	0.269
Alb (g/dL)	3.5 (3.0–4.2)	3.6 (2.5–4.9)	0.225
ChE (U/L)	199 (103–321)	228 (190–553)	0.443
T-cho (mg/dL)	156 (99–207)	180 (67–472)	0.160
T.bil (mg/dL)	0.9 (0.5–3.1)	0.8 (0.3–5.8)	0.412
Cr (mg/dL)	0.62 (0.48–0.88)	0.74 (0.29–7.68)	0.148
CRP	0.26 (0.04–2.38)	0.23 (0.01–18.1)	0.892
CA19-9 (U/mL)	470 (4–4207)	43 (1–24,164)	0.009
DUPAN-2	160 (56–1800)	110 (24–140,000)	0.176
Surgery-related factors			
Surgical procedure,* n* (%)			0.527
Hepatectomy with BDR	5 (45.5%)	90 (40.9%)	
Hepatectomy	2 (18.2%)	23 (10.5%)	
PD	2 (18.2%)	71 (32.3%)	
Cholecystectomy* with BDR	2 (18.2%)	19 (8.6%)	
Cholecystectomy*	0 (0%)	17 (7.7%)	
Concomitant vascular resection			
HAR,* n* (%)	1 (9.1%)	20 (9.1%)	1.000
PVR,* n* (%)	2 (18.2%)	25 (11.4%)	0.492
Operation time (min) (median, range)	349 (263–474)	368 (70–752)	0.485
Blood loss (mL) (median, range)	342 (150–940)	680 (1–6374)	0.075
Blood transfusion, *n* (%)	3 (27.3%)	63 (28.6%)	0.807
Complication (grade ≥ 3*), *n* (%)	3 (27.3%)	62 (28.2%)	0.781
In-hospital death, *n* (%)	0 (0%)	9 (4.1%)	0.494
Pathological findings			
Histology well / mod / poor / other, *n*	3/7/1/0	80/100/23/17	0.521
T3 or T4, *n*	9 (81.8%)	80 (36.4%)	0.003
Lymph node metastasis,* n* (%)	8 (72.7%)	90 (40.9%)	0.037
Number of metastatic LN (median, range)	2 (0–28)	0 (0–11)	0.018
M1,* n* (%) PALN Lymph node* Liver	3 (27.3%) 3 (27.3%) 0 (0%) 0 (0%)	18 (8.2%) 15 (6.8%) 2 (0.9%) 1 (0.5%)	0.107 0.058 0.751 0.823
Residual tumor,* n* (%)			
R0 R1 R2	4 (36.4%) 7 (63.6%) 0 (0%)	161 (73.2%) 57 (25.9%) 2 (0.9%)	0.021

*PWC* peritoneal washing cytology; *BMI* body mass index; *ICC* intrahepatic cholangiocarcinoma; *GBC* gall bladder carcinoma; *HC* hilar cholangiocarcinoma; *DC* distal cholangiocarcinoma; *EBD* endoscopic biliary drainage; *PTBD* percutaneous transhepatic biliary drainage; *Hb* hemoglobin; *Alb* albumin; *ChE* cholinesterase; *T-cho* total cholesterol; *T.bil* total bilirubin; *Cr* creatinine; *HbA1c* hemoglobin A1c; *CA19-9* serum carbohydrate antigen 19–9; Hepatectomy with BDR*, hepatectomy with extrahepatic bile duct resection (including hepatopancreatoduodenectomy), *PD* pancreaticoduodenectomy; cholecystectomy*, cholecystectomy including concomitant resection of the liver bed; *HAR* hepatic artery resection and reconstruction; *PVR* portal vein resection and reconstruction; well / mod / poor /, well, moderately, poorly differentiated adenocarcinoma; *PALN* para-aortic lymph node; Lymph node*, lymph node metastasis on the posterior aspect of the head of the pancreas. It is considered as M1 in patients with left-sided ICC

**Table 2:** Postoperative courses of the positive and negative peritoneal washing cytology groups

	Positive PWC (*n* = 11)	Negative PWC (*n* = 220)	*P* value
Adjuvant chemotherapy,* n* (%)	10 (90.9%)	156 (70.9%)	0.273
Recurrence,* n* (%)	9 (81.8%)	97 (44.1%)	0.014
Initial recurrence site,* n* (%)			
Peritoneum Local Liver Lung Lymph node Others	6 (54.5%) 1 (9.1%) 2 (18.2%) 0 (0%) 1 (9.1%) 1 (9.1%)	20 (9.1%) 32 (14.5%) 31 (14.1%) 14 (6.4%) 25 (11.4%) 9 (4.1%)	0.017 0.950 0.950 0.829 0.798 0.513
Survival after recurrence (months) (median)	9.6	10.7	0.231

*PWC* peritoneal washing cytology

**Table 3:** Clinical course of patients in the positive peritoneal washing cytology group

Case	Disease	Age	Sex	CA19-9	UICC Stage	Adjuvant chemotherapy	Initial Recurrence	Time interval to recurrence*	Survival Period*	Outcome
1	ICC	46	F	4207	T2N2M1* Stage 4	GS	Local	11 m	16 m	D (BTC)
2	ICC	71	F	506	T4N0M0 Stage 3	GS	Bone	7 m	10 m	D (BTC)
3	ICC	51	F	18	T3N1M1* Stage 4	GS	Liver	24 m	37 m	D (BTC)
4	HC	70	M	734	T3N0M0 Stage 3	GS	Peritoneum	14 m	31 m	D (BTC)
5	HC	71	M	470	T3N1M0 Stage 3	GS	Peritoneum	29 m	49 m	A (Recurrence)
6	HC	74	F	3489	T3N1M0 Stage 3	None	Peritoneum, LN	3 m	4 m	D (BTC)
7	HC	77	F	92	T3N1M0 Stage 3	GS	None	None	8 m	D (other)
8	GBC	74	F	764	T3N1M1* Stage 4	GS	Peritoneum, Liver	3 m	11 m	D (BTC)
9	GBC	85	M	186	T3N0M0 Stage 3	S1	Peritoneum	6 m	23 m	D (BTC)
10	DC	68	M	81	T3N2M0 Stage 3	GS	Peritoneum	5 m	16 m	D (BTC)
11	DC	78	F	4	T2N1M0 Stage 2	GS	None	None	141 m	A (RFS)

*CA 19–9*, carbohydrate antigen 19–9 value at the preoperative period (U/ml); Time interval to recurrence*, months from surgery to recurrence; Survival period*, survival period after surgery; *ICC* intrahepatic cholangiocarcinoma; *HC* hilar cholangiocarcinoma; *GBC* gall bladder carcinoma; *DC* distal cholangiocarcinoma; *M* male; *F* female; *EBD* endoscopic biliary drainage; *PTBD* percutaneous transhepatic biliary drainage; M1* metastasis to para-aortic lymph node; *GS* biweekly gemcitabine & S1; *LN* lymph node; *m* months; *D (BTC)* dead by biliary tract cancer; *D* (other), dead by other disease, *A* (Recurrence), alive with recurrence; *A* (RFS), alive (recurrence-free survival)

**Table 4:** Preoperative factors independently associated with positive peritoneal washing cytology

Factors	Odds ratio*	*P* value
CA19-9 (> 80 U/mL)	5.84 (1.17 – 29.0)	0.031
T3 or T4	4.35 (0.87–22.0)	0.074
Number of lymph node metastasis (≥ 2)	5.28 (1.28 – 21.8)	0.021

*CA19-9* serum carbohydrate antigen 19–9; Odds ratio*, numbers in parentheses display 95% confidence interval

### Statistical analysis

A normality test could not verify the normality of the data. Therefore, median values and nonparametric statistical testing procedures were utilized. Patients alive in December 2022 were censored at the time of follow-up. Survival curves were established using the Kaplan–Meier method. The diagnostic value for positive PWC was assessed by calculating the area under the receiver operating characteristic (ROC) curve. Factors independently associated with positive PWC were investigated using logistic regression analysis. All statistical analyses were performed using JMP statistical software version 13 (SAS Institute, Cary, NC). A p value of < 0.05 indicated statistical significance.

## Results

### Eligible patients

Two hundred and ninety-four patients with BTC underwent curative-intent surgery between March 2009 and December 2021. Among them, ten patients without PWC were excluded (Fig. 1). Fifty-three patients with AC were excluded according to the previous multi-institutional retrospective study [19]. All patients with AC showed negative PWC. The remaining 231 patients were eligible for this study. Eleven (4.8%) patients exhibited positive PWC status, and the remaining 220 (95.2%) patients had negative PWC status.

### Clinicopathological features of the positive and negative peritoneal washing cytology groups

The clinicopathological features of eligible patients are shown in Table 1. Except for preoperative CA19-9 values, preoperative factors and surgery-related factors showed no significant differences between the two groups. CA19-9 values were significantly higher in the positive PWC group (*p* = 0.009). Regarding pathological findings, T3 or T4 cancers were significantly more common in the positive PWC group than in the negative PWC group (81.8% *vs.* 40.9%, *p* = 0.003). The ratio of lymph node metastasis and the number of metastatic lymph node were significantly greater in the positive PWC group (*p* = 0.037, *p* = 0.018, respectively). M1 cases were more common in the positive PWC group (27.3%* vs.* 8.2%), although the difference didn’t show statistical significance (*p* = 0.107). Most of the sites of distant metastases were para-aortic lymph nodes in M1 cases. R0 resection rates were 36.4% and 73.2% in the positive and negative PWC groups, and R1 resection rates were 63.6% and 25.9%, respectively. R1 resection rate was significantly higher in the positive PWC group (*p* = 0.021).

### Postoperative courses of the positive and negative peritoneal washing cytology groups

Ten (90.9%) patients in the positive PWC group and 156 (70.9%) in the negative PWC group received adjuvant chemotherapy (*p* = 0.273) (Table 2). Recurrence was significantly more common in the positive PWC group than that in the negative PWC group (81.8% *vs*. 44.1%,* p* = 0.014). The comparison of initial recurrence site between the groups showed that peritoneal recurrence was significantly more common in the positive PWC group than that in the negative PWC group (54.5% *vs.* 9.1%, *p* = 0.017). The ratio of local recurrence showed no significant difference between the two groups (9.1% *vs.* 14.5%, *p* = 0.950). The survival period after recurrence showed no significant difference (median period: 9.6 *vs.* 10.7 months, *p* = 0.231).

### Clinical course of patients in the positive peritoneal washing cytology group

Serum CA19-9 values were elevated in 9 (81.8%) of 11 patients in the positive PWC group (Table 3). Ten (90.9%) patients were diagnosed with stage 3 or 4 BTCs, although the criteria were different in each BTC. Six (54.5%) patients showed early recurrence within postoperative 1 year. Only 1 patient achieved long survival without recurrence for postoperative 141 months.

### Recurrence-free survival

The median RFS time was 12.0 and 60.7 months in the positive and negative PWC groups, and it was significantly shorter in the positive PWC group (*p* = 0.005) (Fig. 2a). The 1-, 2-, and 5-year RFS rates were 43.6%, 32.7%, and 21.8% in the positive PWC group, and 80.0%, 63.2%, and 50.1% in the negative PWC group, respectively.

Eight patients in the positive PWC group and 202 patients in the negative PWC group were M0 cases (Table 1). Among M0 cases, the median RFS time was 10.8 and 87.8 months in the positive and negative PWC groups, and it was significantly shorter in the positive PWC group (*p* = 0.034) (Fig. 3a). The 1-, 2-, and 5-year RFS rates were 50.0%, 33.3%, and 33.3% in the positive PWC group, and 82.1%, 66.3%, and 54.3% in the negative PWC group, respectively.

### Overall survival

The median follow-up for all patients was 31.8 months. Eight (72.7%) of 11 patients in the positive PWC group died of primary cancer and one (9.1%) died of other disease during follow-up. Eighty-one (36.8%) of 220 patients in the negative PWC group died of primary cancer and 22 (10.0%) died of other diseases during follow-up. The median survival time was 17.0 and 60.6 months in the positive and negative PWC groups, and it was significantly shorter in the positive PWC group (*p* = 0.008) (Fig. 2b). The 1-, 2-, and 5-year survival rates were 63.6%, 36.4%, and 18.2% in the positive PWC group, and 88.0%, 74.7%, and 50.6% in the negative PWC group, respectively.

Among M0 cases, the median survival time was 20.2 and 64.7 months in the positive and negative PWC groups, and it was significantly shorter in the positive PWC group (*p* = 0.041) (Fig. 3b). The 1-, 2-, and 5-year survival rates were 62.5%, 37.5%, and 25.0% in the positive PWC group, and 91.0%, 77.6%, and 54.1% in the negative PWC group, respectively.

### Recurrence-free survival and overall survival in each carcinoma type

No significant differences in RFS existed between the four carcinomas (Supplemental Fig. 1a). No significant differences in OS existed between the four carcinomas (Supplemental Fig. 1b).

### Factors independently associated with positive PWC

Multivariate analyses were performed using the three indices (CA19-9 value, T3 or T4 cancer, and number of lymph node metastasis), which were estimated to be predictive of positive PWC in clinicopathological features. Prior to the multivariate analysis, the optimal cut-off values for positive PWC were assessed by ROC curves and AUCs. The AUC was 0.705 for preoperative CA19-9 value with an optimal cut-off value of 81 U/ml, which yielded 81.8% sensitivity and 57.3% specificity. The AUC was 0.718 for the number of lymph node metastasis with an optimal cut-off value of 2.0, which yielded 72.3% sensitivity and 74.5% specificity. Multivariate analysis was performed using these cut-off values. Serum CA 19–9 level over 80 U/mL and multiple lymph node metastasis were independently associated with positive PWC (odds ratio [OR]: 5.84, *p* = 0.031; OR: 5.28, *p* = 0.021, respectively).

## Discussion

The clinical importance of PWC for BTC patients has scarcely elucidated due to a lack of previous investigations. To our knowledge, only a few studies have described PWC for BTC [17–19]. Martin et al. first reported the results of PWC obtained before planned open surgery in 26 patients with HC in 2001, and positive PWC was confirmed in two [17]. However, both patients had gross peritoneal metastases; therefore, the prognostic impact of positive PWC status without other inoperable factors was unclear. Ajki et al. reported the results of PWC obtained at the beginning of laparotomy in 41 patients with BTCs, and the overall incidence of positive PWC was 9.8% [18]. The prevalence of positive PWC in the TNM staging system were 0% in T1/T2, 6% in T3, 38% in T4, 0% in N0, 25% in N1, 3% in M0, 27% in M1, respectively. Positive PWC has a tendency to be found in more advanced BTCs [18]. Similarly, T3 or T4 cancers were significantly more common in the positive PWC group in our study. The largest previous study was the Kansai Hepato-Biliary Oncology (KHBO) 1701 study in Japan. The study reported that five (3.0%) patients showed positive PWC among 169 patients who underwent R0 resection [19].

Regarding type of carcinoma, 3 (7.3%) of 41 ICC, 2 (3.6%) of 55 GBC, 4 (5.6%) of 72 HC, 2 (3.2%) of 63DC, and none (0%) of 53 AC patients showed positive PWC. Similarly, none of the 48 patients with AC had positive PWC in the KHBO 1701 study, suggesting that patients with AC are less likely to have positive PWC.

The overall incidence of positive PWC was 4.8% in our study, which was higher than the 3.0% reported in the KHBCO 1701 study. The KHBO group included only patients who received R0 resection and excluded patients with R1 resection. If eligible patients were confined to 165 patients with R0 resection in our study, only four (2.4%) patients had positive PWC. Sixty-four patients received R1 resection in our study, and among them, seven (10.9%) patients had positive PWC. The rate of positive PWC was significantly higher in patients with R1 resection compared to those with R0 resection. However, preoperatively distinguishing between R0 and R1 resection is impossible, and therefore, we decided to include R1 resection cases in this study.

Regarding postoperative recurrence, peritoneal recurrence was significantly more common in the positive PWC group in both the KHBO 1701 study and our study. Several reports have described disseminating cancer cells in the peritoneal cavity after preoperative percutaneous transhepatic biliary drainage (PTBD) [23–28]. In our study, one of 5 patients who had received preoperative PTBD showed positive PWC status. Although it was unclear whether PTBD had induced positive PWC status in this patient, preoperative PTBD should be refrained as possible considering the risk of peritoneal dissemination.

Regarding the prognostic impact of positive PWC status, the RFS and overall survival times in the positive PWC group were significantly shorter than those in the negative PWC group. Except for one patient who died from another disease, six of 10 patients with positive PWC had early recurrences within postoperative 1 year (Table 3), and the median survival period was as short as 17.0 months. Further, among M0 cases, the RFS and overall survival times in the positive PWC group were significantly shorter than those in the negative PWC group. These results indicates that the positive PWC status alone can be a poor prognostic factor, and patients in the positive PWC group may not gain prolonged survival from surgery. To identify patients with positive PWC before curative-intent surgery, minimally invasive staging laparotomy (SL) may be optimal [29–31]. However, with the low incidence of positive PWC, it is difficult to perform SL in all patient with BTC. Therefore, patients with preoperative CA19-9 value over 80 U/mL might be the candidate for SL. Nine (10.7%) of 84 patients with preoperative CA19-9 value over 80 U/mL showed positive PWC in this study. The multiple lymph node metastasis was also independently associated with positive PWC status. However, preoperative diagnosis of lymph node metastasis is difficult, because BTCs are often accompanied with cholangitis and subsequent inflammatory lymphadenopathy. Although it remains unclear whether positive PWC status is equivalent to M1 or not, preoperative SL in patients with high CA19-9 level may be useful to eliminate invalid surgery by detecting positive PWC.

This study has some limitations. First, this study was based on data from a single center’s database, and the unexpected bias cannot be completely excluded. Second, positive PWC in BTC is a rare occurrence, and the number of patients with positive PWC is small. Future studies using data from multiple centers will be necessary to completely prove the clinical impact of positive PWC status. Third, the paradox of median RFS time and OS time was found in the negative PWC group. In the negative PWC group, 103 patients died during follow-up, and among them, 22 (21.3%) patients died without recurrence of BTC. On the other hand, in the positive PWC group, nine patients died during follow-up, and among them, 1 (11.1%) patient died without recurrence. The relatively high rate of death without recurrence was surmised to induce this paradox in the negative PWC group.

In conclusion, patients with positive PWC showed earlier recurrence and shorter survival compared with those with negative PWC.

### Supplementary Information

Below is the link to the electronic supplementary material.

Supplementary file1 (JPG 89 KB)

## Data Availability

The data are not publicly available due to their containing information that could compromise the privacy of research participants.
