# Supplementary figures and images for: Prognostic impact of peritoneal washing cytology in patients with biliary tract cancer
Source: Langenbecks Arch Surg. 2024 Jan 22;409(1):45. doi: 10.1007/s00423-024-03233-y (PMC10803468; doi:10.1007/s00423-024-03233-y)

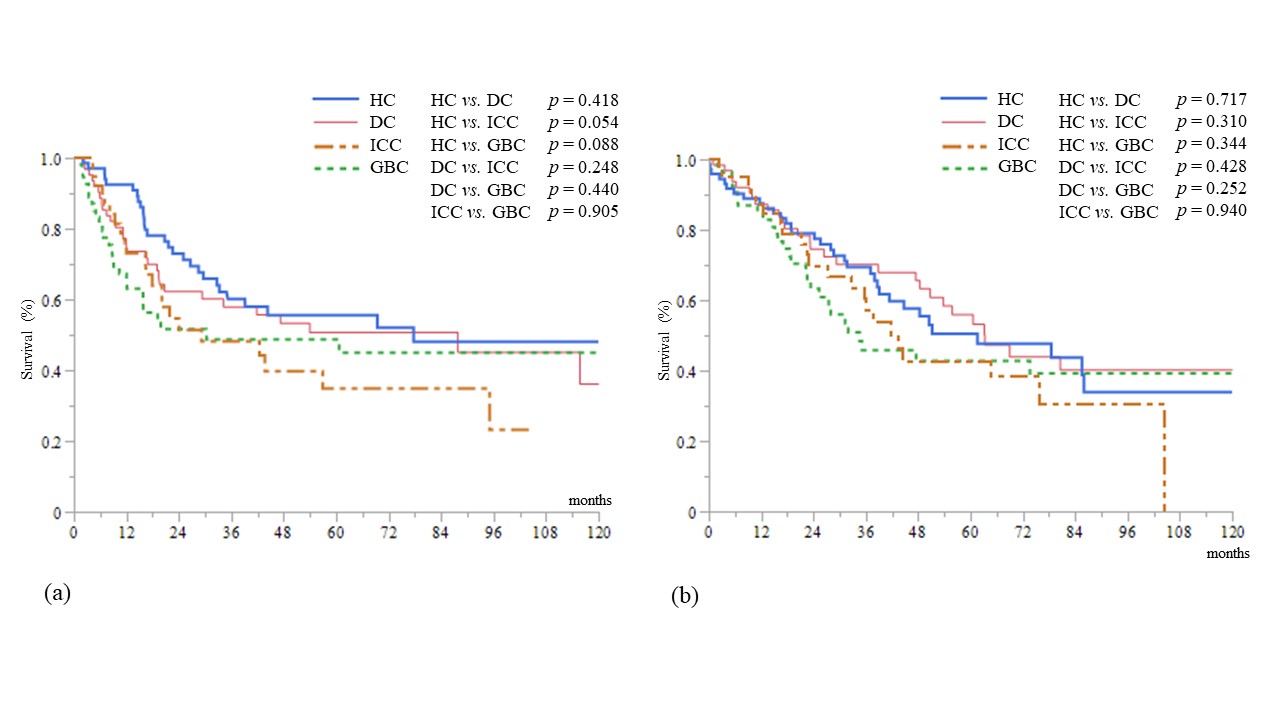

Supplement: Supplementary file 1 — Supplementary file1 (JPG 89 KB) [file 423_2024_3233_MOESM1_ESM.jpg]
